# Measures of the coupling between fluctuating brain network organization and heartbeat dynamics

**DOI:** 10.1162/netn_a_00369

**Published:** 2024-07-01

**Authors:** Diego Candia-Rivera, Mario Chavez, Fabrizio De Vico Fallani

**Affiliations:** Sorbonne Université, Paris Brain Institute (ICM), CNRS UMR 7225, INRIA Paris (Nerv Team), INSERM U1127, AP-HP Hôpital Pitié-Salpêtrière, Paris, France

**Keywords:** Brain-heart interplay, Network physiology, Parkinson’s disease, Dopamine, Brain connectivity, Cardiac sympathetic-parasympathetic activity

## Abstract

In recent years, there has been an increasing interest in studying brain–heart interactions. Methodological advancements have been proposed to investigate how the brain and the heart communicate, leading to new insights into some neural functions. However, most frameworks look at the interaction of only one brain region with heartbeat dynamics, overlooking that the brain has functional networks that change dynamically in response to internal and external demands. We propose a new framework for assessing the functional interplay between cortical networks and cardiac dynamics from noninvasive electrophysiological recordings. We focused on fluctuating network metrics obtained from connectivity matrices of EEG data. Specifically, we quantified the coupling between cardiac sympathetic–vagal activity and brain network metrics of clustering, efficiency, assortativity, and modularity. We validate our proposal using open-source datasets: one that involves emotion elicitation in healthy individuals, and another with resting-state data from patients with Parkinson’s disease. Our results suggest that the connection between cortical network segregation and cardiac dynamics may offer valuable insights into the affective state of healthy participants, and alterations in the network physiology of Parkinson’s disease. By considering multiple network properties, this framework may offer a more comprehensive understanding of brain–heart interactions. Our findings hold promise in the development of biomarkers for diagnostic and cognitive/motor function evaluation.

## INTRODUCTION

There is strong clinical evidence indicating that various cardiovascular, neurological, and psychiatric disorders can affect brain–heart interactions (Samuels, 2007; Silvani, Calandra-Buonaura, Dampney, & Cortelli, 2016). These interactions are involved in multiple bodily processes, including sensing, integration, and regulation of activity to maintain homeostatic balance (Craig, 2002). The brain–heart communication is bidirectional and occurs through different neural mechanisms, such as the vagal and spinal pathways (Chen et al., 2021). Experimental findings demonstrate that these afferent and efferent communications have a significant impact on perception, information processing, action, and spontaneous cognition (Azzalini, Rebollo, & Tallon-Baudry, 2019; Candia-Rivera, 2022; Skora, Livermore, & Roelofs, 2022).

Recent advancements in neuroscience have highlighted the importance of adopting an embodied perspective when investigating brain function (Chen et al., 2021; Quigley, Kanoski, Grill, Barrett, & Tsakiris, 2021), some with a particular emphasis on studying the brain–heart communication (Candia-Rivera, 2022; Valenza, Toschi, & Barbieri, 2016). These interactions have traditionally been studied through the analysis of heartbeat-evoked responses (Coll, Hobson, Bird, & Murphy, 2021; H.-D. Park & Blanke, 2019), that is, brain activity locked to the cardiac cycle. Various methods have been developed to explore the relationship between brain oscillations and the autonomic nervous system, such as signal processing techniques that analyze correlation, directional coupling, co-occurrences, or phase synchronization (Candia-Rivera, Catrambone, & Valenza, 2021). However, previous studies have predominantly focused on the interaction between specific brain or scalp regions and heartbeat dynamics, disregarding the dynamic nature of brain networks and their role in numerous cognitive functions (Bashan, Bartsch, Kantelhardt, Havlin, & Ivanov, 2012; Bressler & Menon, 2010; Faes et al., 2022; H.-J. Park & Friston, 2013).

In this article, we propose a new framework for studying brain–heart interactions that explores the relationship between ongoing brain network organization and cardiac sympathetic–vagal oscillations. Our framework goes beyond state-of-the-art approaches (Candia-Rivera, Catrambone, & Valenza, 2021), which typically describe the relationship between a single brain region and heartbeat dynamics. Instead, it provides biomarkers related to large-scale brain–heart interaction by quantifying the intricate dynamics between global brain activity and cardiac dynamics. This approach may be useful for understanding certain conditions; for instance, in Parkinson’s disease, global brain dynamics are impacted because of the neural damage caused in several parts of the nervous system (Candia-Rivera, Vidailhet, Chavez, & De Vico Fallani, 2024; Hammond, Bergman, & Brown, 2007). Additionally, Parkinson’s disease causes the emergence of parallel autonomic symptoms (Sharabi, Vatine, & Ashkenazi, 2021). However, these physiological changes may not necessarily serve as definitive hallmarks for characterizing the disease. This uncertainty arises from the high heterogeneity of Parkinson’s disease and the lack of reliability of these biomarkers, which is, in turn, rooted in our limited understanding of their underlying mechanisms (Palma & Kaufmann, 2014). This further underscores the need to explore global brain dynamics in order to understand the extent to which brain–heart interaction measurements can uncover specific aspects of the disease (Iniguez et al., 2022).

In the realm of affective processing, brain–heart interactions have been previously described in the role of visceral activity in arousal (Candia-Rivera, Catrambone, Thayer, Gentili, & Valenza, 2022; Hsueh et al., 2023; Klein, Dolensek, Weiand, & Gogolla, 2021). Our framework may serve to further explore the role of heartbeat dynamics in affective processing, for instance, to unravel the intricate components defining affective states, such as valence and dominance, whose neural correlates remain to be uncovered and may likely correspond to large-scale neural dynamics (Lindquist, Satpute, Wager, Weber, & Barrett, 2016). Our framework serves as a proof-of-concept, showcasing how the study of brain–heart interactions can be approached in various conditions where global neural dynamics are not fully understood by solely examining the dynamics of specific brain regions.

We test our framework in two openly available EEG/ECG datasets: one on emotion elicitation in 32 healthy participants (Koelstra et al., 2012) and another that includes 15 Parkinson’s disease patients in a resting state (George et al., 2013). We delved into the variations in global network dynamics, focusing on parameters such as efficiency, clustering, modularity, and assortativity within EEG connectivity matrices. Our investigation aimed to understand the connection between these dynamics and the fluctuations in cardiac sympathetic–vagal dynamics. The conditions we examined included comparisons between resting states and emotion elicitation, as well as differences between the resting states of healthy individuals and those with Parkinson’s disease. Additionally, we investigated the influence of dopamine medication on the resting state of individuals with Parkinson’s disease. This intervention is designed to pharmacologically replenish the disrupted dopamine levels resulting from the loss of dopamine-producing cells. Furthermore, we investigated how changes in brain–heart coupling relate to alterations in motor symptoms when dopamine medication is used.

The application of our framework to study the interaction between brain networks and cardiac activity in Parkinson’s disease revealed significant potential. The development of new frameworks to understand large-scale brain–heart interactions may provide a more comprehensive understanding of the functional brain–heart connection and may offer valuable insights into the role of these interactions in health and disease.

## RESULTS

**Figure 1.**
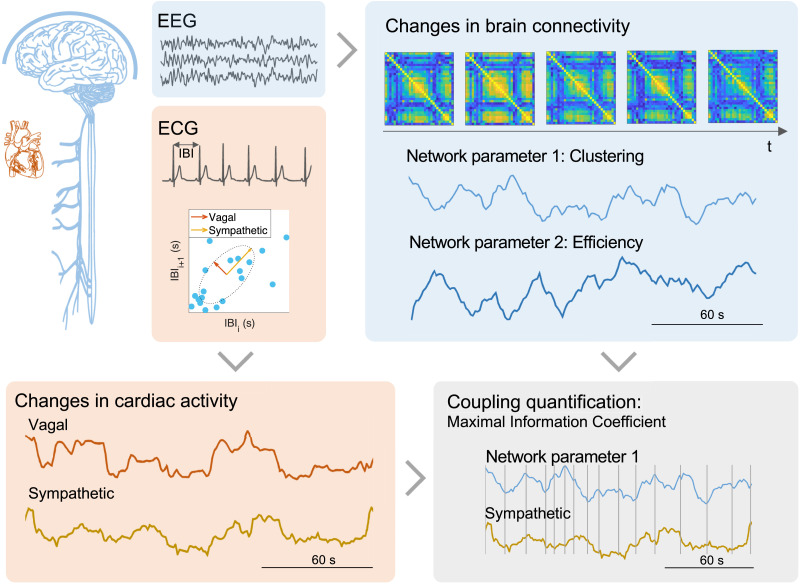
Brain network–cardiac coupling framework. The framework aims to estimate the coupling between brain network metrics and cardiac autonomic dynamics. Estimation of cardiac sympathetic–vagal activity is computed from the successive changes in interbeat intervals (IBI) gathered from the ECG, and estimation of the fluctuating network metrics is computed from connectivity matrices per each frequency band of the EEG. The coupling quantification is achieved by assessing the similarities between two time series, regardless of the curvature of the signals. The maximal information coefficient (MIC) method evaluates similarities between distinct segments individually, using an adjusted grid as depicted in the figure. The overall measure combines the similarities observed throughout the entire time course.

We examined brain network metrics derived from EEG data, as well as cardiac sympathetic–vagal activity obtained from ECG recordings in two datasets: one including healthy participants undergoing emotion elicitation, and another including patients with Parkinson’s disease during resting state. Brain connectivity matrices were computed from the EEG time-frequency representations and consecutive coherence computation for all pairs of EEG channels, separately for alpha, beta, and gamma bands (Cattai et al., 2021). Then, connectivity matrices were binarized using a efficiency–cost optimization algorithm (De Vico Fallani, Latora, & Chavez, 2017) to finally compute network metrics, including clustering, efficiency, modularity, and assortativity (Rubinov & Sporns, 2010). Cardiac sympathetic–vagal activity was assessed using a method that quantified successive changes in the interbeat intervals (IBI). To distinguish between slow and fast changes in IBI over time, we employed Poincaré plots. These changes are proposed to be indicative of cardiac sympathetic and vagal activities, respectively (Candia-Rivera, 2023). The coupling between brain network metrics and cardiac activity was estimated using the maximal information coefficient (MIC) method (Reshef et al., 2011), an alternative method to the standard correlation coefficient accounting for potential nonlinearities of the signals. A general scheme of our approach is displayed in Figure 1.

We computed time-varying values of network clustering, efficiency, assortativity, and modularity from EEG activities in the alpha (*α*), beta (*β*), and gamma (*γ*) bands. The changes in brain–heart coupling, as estimated with MIC values, are presented in the four possible comparisons: resting state versus emotion elicitation in healthy participants; and healthy participants versus Parkinson’s disease patients, at rest (Table 1). We compared Parkinson’s disease patients under their regular medications (on dopamine medication), and patients under at least 12 hr of suspended medications (off dopamine medication).

**Table 1.** Results on dopamine medication in Parkinson’s disease patients. Statistics correspond to the Wilcoxon tests on the changes in the coupling between network metrics (in alpha, beta, and gamma bands) and cardiac sympathetic–vagal dynamics, on- versus off-dopamine medication conditions. **Bold** indicates significance (*p* < 0.05, which was confirmed by a permutation test); *p* < 0.001 indicates that none of the 1,000 random permutations surpassed the effect magnitude from the original samples. *Z* > 0 indicates Condition 2 > Condition 1.

Heart–brain coupling feature			Rest vs. Emotion	PD on vs. PD off	HS vs. PD off	HS vs. PD on
Cardiac sympathetic	Alpha network	Clustering	***p* < 0.001, *Z* = 3.7211**	***p* = 0.032, *Z* = 2.2151**	*p* = 0.5936, *Z* = 0.5336	*p* = 0.2280, *Z* = −1.2056
		Efficiency	***p* < 0.001, *Z* = 3.5715**	*p* = 0.6496, *Z* = 0.4544	*p* = 0.7073, *Z* = 0.3755	*p* = 0.8279, *Z* = −0.2174
		Assortativity	***p* < 0.001, *Z* = 3.7211**	*p* = 0.4603, *Z* = 0.7384	*p* = 0.5401, *Z* = −0.6127	*p* = 0.2131, *Z* = −1.2451
		Modularity	***p* < 0.001, *Z* = 3.4220**	*p* = 0.6909, *Z* = −0.3976	***p* = 0.0420, *Z* = −2.1148**	*p* = 0.4177, *Z* = −0.8103
	Beta network	Clustering	***p* < 0.001, *Z* = 3.4406**	*p* = 0.0783, *Z* = −1.7607	*p* = 0.9842, *Z* = 0.0198	*p* = 0.0855, *Z* = 1.7195
		Efficiency	***p* = 0.001, *Z* = 3.0853**	*p* = 0.5321, *Z* = 0.6248	*p* = 0.7073, *Z* = 0.3755	*p* = 0.8900, *Z* = −0.1383
		Assortativity	***p* < 0.001, *Z* = 3.9642**	*p* = 0.0995, *Z* = −1.6471	*p* = 0.1854, *Z* = −1.3242	*p* = 0.3529, *Z* = 0.9289
		Modularity	***p* < 0.001, *Z* = 3.7772**	*p* = 0.0609, *Z* = 1.8743	*p* = 0.2436, *Z* = 1.1661	*p* = 0.1491, *Z* = −1.4428
	Gamma network	Clustering	***p* < 0.001, *Z* = 3.7772**	***p* = 0.001, *Z* = 3.0102**	*p* = 0.1280, *Z* = 1.5218	*p* = 0.0604, *Z* = −1.8776
		Efficiency	***p* = 0.002, *Z* = 2.9170**	***p* = 0.001, *Z* = 2.9534**	***p* = 0.0410, *Z* = 2.1543**	*p* = 0.3738, *Z* = −0.8894
		Assortativity	***p* = 0.003, *Z* = 3.2536**	*p* = 1, *Z* = 0	*p* = 0.3328, *Z* = −0.9684	*p* = 0.2436, *Z* = −1.1661
		Modularity	***p* < 0.001, *Z* = 3.4032**	*p* = 0.3942, *Z* = 0.8519	*p* = 0.7369, *Z* = −0.3360	*p* = 0.3135, *Z* = −1.0080
Cardiac vagal	Alpha network	Clustering	***p* < 0.001, *Z* = 3.3284**	*p* = 0.4603, *Z* = 0.7384	*p* = 0.9527, *Z* = −0.0593	*p* = 0.3328, *Z* = −0.9684
		Efficiency	***p* < 0.001, *Z* = 4.4129**	*p* = 0.3942, *Z* = 0.8519	*p* = 0.9213, *Z* = 0.0988	*p* = 0.7669, *Z* = −0.2965
		Assortativity	***p* < 0.001, *Z* = 4.0764**	*p* = 0.8203, *Z* = −0.2272	*p* = 0.5143, *Z* = −0.6522	*p* = 0.7369, *Z* = −0.3360
		Modularity	***p* < 0.001, *Z* = 4.5251**	*p* = 0.4955, *Z* = 0.6816	*p* = 0.5401, *Z* = 0.6127	*p* = 0.9213, *Z* = −0.0988
	Beta network	Clustering	***p* < 0.001, *Z* = 4.1699**	*p* = 0.3343, *Z* = −0.9655	*p* = 0.2436, *Z* = −1.1661	*p* = 0.8279, *Z* = −0.2174
		Efficiency	***p* = 0.005, *Z* = 2.7861**	*p* = 0.8203, *Z* = −0.2272	*p* = 0.3738, *Z* = 0.8894	*p* = 0.6637, *Z* = 0.4349
		Assortativity	***p* < 0.001, *Z* = 3.7959**	*p* = 0.6496, *Z* = −0.4544	*p* = 0.7972, *Z* = −0.2569	*p* = 0.9213, *Z* = 0.0988
		Modularity	***p* = 0.001, *Z* = 3.3471**	*p* = 0.0783, *Z* = 1.7607	*p* = 0.9527, *Z* = −0.0593	*p* = 0.1009, *Z* = −1.6404
	Gamma network	Clustering	***p* < 0.001, *Z* = 4.3382**	*p* = 0.1728, *Z* = 1.3631	*p* = 0.9842, *Z* = 0.0198	*p* = 0.1989, *Z* = −1.2847
		Efficiency	***p* < 0.001, *Z* = 3.9648**	*p* = 1, *Z* = 0	*p* = 0.5665, *Z* = −0.5732	*p* = 0.7669, *Z* = −0.2965
		Assortativity	***p* < 0.001, *Z* = 3.7211**	*p* = 0.2805, *Z* = −1.0791	*p* = 0.2436, *Z* = −1.1661	*p* = 0.6212, *Z* = −0.4941
		Modularity	***p* = 0.010, *Z* = 2.6552**	*p* = 0.6496, *Z* = −0.4544	*p* = 0.6494, *Z* = −0.4546	*p* = 0.9527, *Z* = 0.0593

Results on emotion elicitation show a strong and generalized change of the brain–heart interaction markers, indicating that the affective state causes a modulation to brain network–cardiac couplings. In Parkinson’s disease patients, the comparison between on- and off-dopamine medication conditions was significant when comparing gamma clustering-sympathetic, gamma efficiency-sympathetic couplings and alpha clustering-sympathetic. The gamma clustering-sympathetic coupling was larger in the off-dopamine medication condition (paired Wilcoxon test, *p* = 0.001, *Z* = 3.0102), as well as in the gamma efficiency-sympathetic coupling (paired Wilcoxon test, *p* = 0.001, *Z* = 2.9534) and in the alpha clustering-sympathetic coupling (paired Wilcoxon test, *p* = 0.032, *Z* = 2.2151). The changes in the coupling were not significant for assortativity and modularity, similar to the comparisons with cardiac vagal activity and in the other frequency bands (Table 1). When comparing Parkinson’s disease patients off dopamine medication with healthy participants, we observed differences in alpha modularity-sympathetic and gamma efficiency-sympathetic couplings. However, when comparing Parkinson’s disease patients on dopamine medication with healthy participants, we did not find any significant differences in the measures we studied.

We performed an additional analysis to control that our measurements of network-cardiac couplings were not influenced exclusively by either the fluctuations of network metrics or cardiac dynamics. We analyzed network metrics separately and controlled whether their means were different when comparing on- against off-dopamine medication conditions (Table 2). It is widely recognized that in certain scenarios, especially those involving emotions, cardiac activity alone can be a reliable predictor. However, this is not always the case. In our study, Parkinson’s disease demonstrated that incorporating brain activity, particularly brain–heart coupling in the gamma frequency range, improves predictive accuracy for the conditions on and off dopamine medication (see the Supporting Information).

**Table 2.** Control tests on the changes in network metrics and cardiac dynamics, separately. **Bold** indicates significance (*p* < 0.05, which was confirmed by a permutation test).

Brain network feature		Rest vs. Emotion	PD on vs. PD off	HS vs. PD off	HS vs. PD on
Alpha network	Clustering	*p* = 0.2950, *Z* = 1.0471	*p* = 0.5321, *Z* = −0.6248	*p* = 0.8279, *Z* = −0.2174	*p* = 0.6781, *Z* = −0.4150
	Efficiency	*p* = 0.5372, *Z* = −0.6171	*p* = 0.2560, *Z* = 1.1359	*p* = 0.5665, *Z* = 0.5732	*p* = 0.3328, *Z* = 0.9684
	Assortativity	*p* = 0.5372, *Z* = 0.6171	*p* = 0.2115, *Z* = 1.2495	*p* = 0.1184, *Z* = 1.5614	*p* = 0.0552, *Z* = 1.9171
	Modularity	*p* = 0.7935, *Z* = −0.2618	*p* = 0.1728, *Z* = 1.3631	*p* = 0.2280, *Z* = 1.2056	*p* = 0.0855, *Z* = 1.7195
Beta network	Clustering	*p* = 0.1608, *Z* = 1.4024	*p* = 0.2805, *Z* = −1.0791	*p* = 1.0000, *Z* = 0.0000	*p* = 0.3738, *Z* = −0.8894
	Efficiency	*p* = 0.1782, *Z* = −1.3463	*p* = 0.4955, *Z* = 0.6816	*p* = 0.5936, *Z* = 0.5336	*p* = 0.1280, *Z* = 1.5218
	Assortativity	*p* = 0.7084, *Z* = −0.3740	*p* = 0.5321, *Z* = −0.6248	*p* = 0.7972, *Z* = 0.2569	*p* = 0.9842, *Z* = 0.0198
	Modularity	*p* = 0.8517, *Z* = 0.1870	*p* = 0.1118, *Z* = 1.5903	*p* = 0.1727, *Z* = 1.3637	*p* = 0.0255, *Z* = 2.2334
Gamma network	Clustering	*p* = 0.9107, *Z* = 0.1122	*p* = 0.1728, *Z* = −1.3631	*p* = 0.3328, *Z* = 0.9684	*p* = 0.8279, *Z* = 0.2174
	Efficiency	*p* = 0.7364, *Z* = −0.3366	*p* = 0.1914, *Z* = 1.3063	*p* = 0.1727, *Z* = −1.3637	*p* = 0.6781, *Z* = −0.4150
	Assortativity	*p* = 0.7364, *Z* = −0.3366	*p* = 0.0609, *Z* = −1.8743	*p* = 0.5401, *Z* = 0.6127	*p* = 0.8588, *Z* = −0.1779
	Modularity	*p* = 0.6268, *Z* = −0.4862	*p* = 0.9096, *Z* = −0.1136	*p* = 0.2436, *Z* = −1.1661	*p* = 0.0661, *Z* = −1.8381
Cardiac features	Sympathetic	***p* = 0.0004, *Z* = 3.5341**	*p* = 0.6909, *Z* = 0.3976	*p* = 0.5143, *Z* = −0.6522	*p* = 0.2770, *Z* = −1.0870
	Vagal	***p* = 0.0016, *Z* = 3.1601**	*p* = 0.5701, *Z* = −0.5680	*p* = 0.3954, *Z* = −0.8499	*p* = 0.8279, *Z* = −0.2174

**Figure 2.**
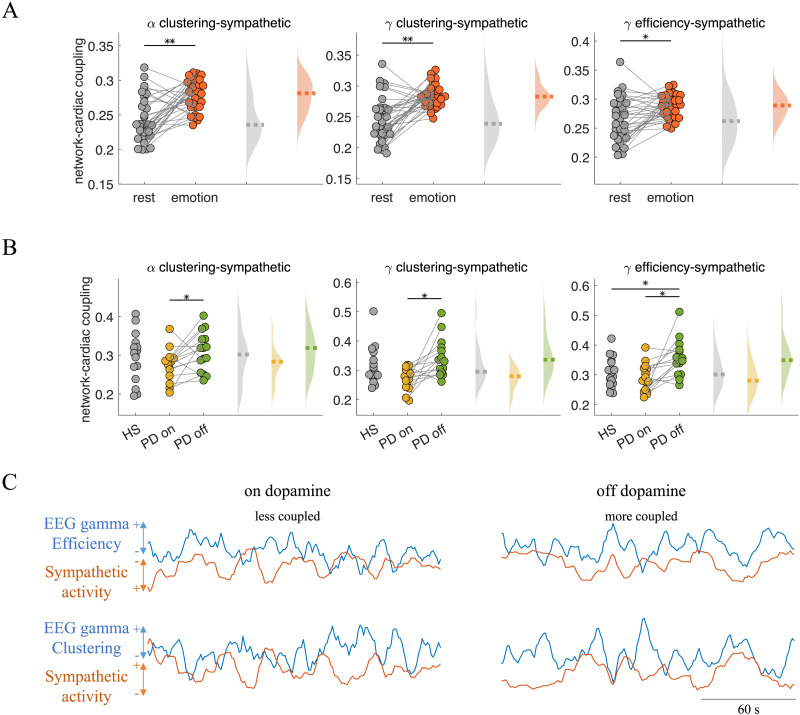
Brain network–cardiac sympathetic couplings. (A, B) Coupling in alpha clustering-sympathetic, in gamma clustering-sympathetic, and in gamma efficiency-sympathetic activity. Individual values correspond to maximal information coefficient (MIC) values. Statistical tests were performed to compare (A) resting state versus emotion elicitation in healthy participants and (B) healthy state (HS) versus Parkinson’s disease (PD) in either on- or off-dopamine medication conditions. Statistical tests were performed using Wilcoxon tests, paired or unpaired as it corresponds. * *p* < 0.05, ** *p* < 0.001, *** *p* < 0.0001. (C) Example of a Parkinson’s disease patient whose network-cardiac coupling changed when comparing on- and off-dopamine medication conditions. Fluctuating network metrics were smoothed with a sliding mean window of 6 s (six samples) for visualization purposes. The amplitude of the displayed signals was scaled for visualization purposes.

Figures 2A and 2B illustrate the brain–heart coupling distribution during emotion elicitation in healthy participants and in resting state in healthy participants and Parkinson’s disease patients. The markers shown represent significant changes observed in Parkinson’s disease patients on versus off dopamine medication, specifically alpha clustering-sympathetic, gamma clustering-sympathetic, and gamma efficiency-sympathetic. Across all conditions investigated, it is evident that the coupling between the brain network and cardiac activity increases during emotion elicitation compared with the resting state. Notably, Parkinson’s disease patients exhibit an increase in this coupling compared with healthy participants when they are off dopamine medication. However, when these patients are on dopamine medication, there are no significant differences observed in this coupling compared with healthy participants. Figure 2C illustrates the covariations in gamma clustering, efficiency, and cardiac sympathetic activity under on- and off-dopamine medication conditions for one patient. In the on-dopamine medication condition, the fluctuations in network metrics are faster and more complex compared with the off-dopamine medication condition. In contrast, under the off-dopamine medication condition there occurs a slowing in the network dynamics, which arises in certain degree of synchrony with cardiac sympathetic dynamics.

**Figure 3.**
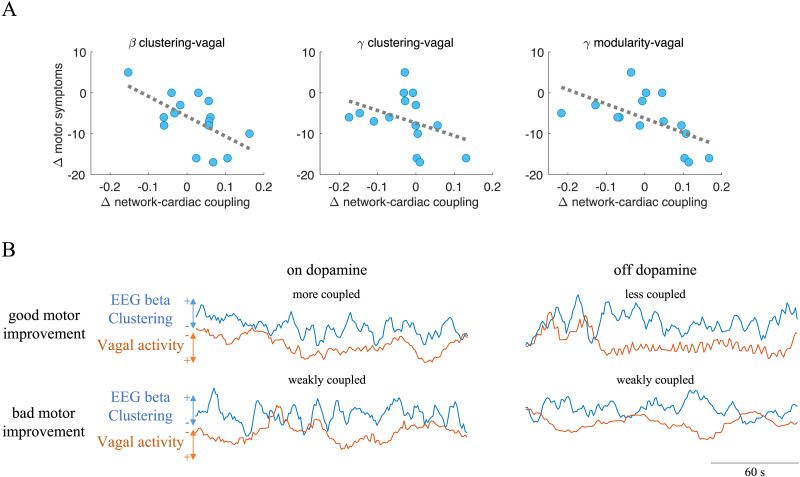
Brain network–cardiac vagal couplings. (A) Spearman correlations between changes in motor symptoms and the changes in network-cardiac coupling, as quantified between on- and off-dopamine medication conditions. (B) Example of a Parkinson’s disease patient with good motor outcome after dopamine medication and another patient with bad motor outcome after dopamine medication. Fluctuations of network metrics were smoothed with a sliding mean window of 6 s (six samples) for visualization purposes. The amplitude of the displayed signals was scaled for visualization purposes.

We further explored whether the changes in on-off dopamine medication in network-cardiac couplings were related to the changes in motor symptoms. The changes in motor symptoms were evaluated by a specialist using the Unified Parkinson’s Disease Rating Scale Part III, UPDRS-III (Movement Disorder Society Task Force on Rating Scales for Parkinson’s Disease, 2003), which ranges from 0 to 132, with severity increasing as numbers go up. We found that the increase in the coupling between cardiac vagal dynamics with different network metrics was related to the improvement in motor symptoms. We found these effects when correlating the improvement in motor symptoms (reduction in the UPDRS-III values) with the cardiac vagal coupling with beta clustering (Spearman correlation, *p* < 0.001, *R* = −0.5717), gamma clustering (Spearman correlation, *p* < 0.001, *R* = −0.5896), and gamma modularity (Spearman correlation, *p* < 0.001, *R* = −0.6559), as shown in Table 3 and Figure 3A.

**Table 3.** Spearman correlation tests on the changes in motor symptoms and the coupling between network metrics (in alpha, beta, and gamma bands) and cardiac sympathetic–vagal dynamics, on versus off conditions. **Bold** indicates significance (*p* < 0.05, which was confirmed by a permutation test); *p* < 0.001 indicates that none of the 1,000 random permutations surpassed the effect magnitude from the original samples.

Heart-brain coupling		ΔClustering	ΔEfficiency	ΔAssortativity	ΔModularity
Cardiac sympathetic	Alpha network	*p* = 0.3033, *R* = −0.2849	*p* = 0.2067, *R* = −0.3459	*p* = 0.9142, *R* = 0.0305	*p* = 0.1180, *R* = −0.4211
	Beta network	*p* = 0.9192, *R* = 0.0287	*p* = 0.5186, *R* = −0.1810	*p* = 0.3161, *R* = 0.2778	*p* = 0.5746, *R* = −0.1577
	Gamma network	*p* = 0.9444, *R* = −0.0197	*p* = 0.2877, *R* = −0.2939	*p* = 0.1944, *R* = −0.3548	*p* = 0.3001, *R* = 0.2867
Cardiac vagal	Alpha network	*p* = 0.9394, *R* = −0.0215	*p* = 0.3001, *R* = −0.2867	*p* = 0.3706, *R* = −0.2491	*p* = 0.4854, *R* = 0.1953
	Beta network	***p* < 0.001, *R* = −0.5717**	*p* = 0.6467, *R* = −0.1290	*p* = 0.1380, *R* = −0.4014	*p* = 0.1582, *R* = −0.3835
	Gamma network	***p* < 0.001, *R* = −0.5896**	*p* = 0.2117, *R* = −0.3423	*p* = 0.9646, *R* = −0.0125	***p* < 0.001, *R* = −0.6559**

Figure 3B illustrates the covariations between the clustering of networks from the beta band and cardiac vagal activity under on- and off-dopamine medication conditions for one patient with good motor outcome and another with bad motor outcome, as quantified with the UPDRS-III. In the on-dopamine medication condition, the fluctuations in beta clustering are more coupled to vagal dynamics, as compared with the off-dopamine medication condition, in the patient with good motor outcome. In contrast, the patient with bad motor outcome did not present this distinction when comparing on- against off-dopamine medication conditions.

Finally, we also controlled that the correlations of network-cardiac couplings with motor outcomes were not influenced exclusively by the fluctuations of network metrics or cardiac activity. We observed a correlation between changes in alpha efficiency and changes in motor symptoms, albeit with lower statistical power compared with the brain–heart coupling measures. However, when analyzing all other network metrics and cardiac activity separately under on- and off-dopamine medication conditions, we did not find any significant correlations with motor symptoms (Table 4).

**Table 4.** Spearman correlation tests on the changes in motor symptoms and network metrics (in alpha, beta, and gamma bands), on versus off conditions. **Bold** indicates significance (*p* < 0.05, which was confirmed by a permutation test).

	Clustering	Efficiency	Assortativity	Modularity
Alpha	*p* = 0.0517, *R* = −0.5108	***p* = 0.0440, *R* = 0.5269**	*p* = 0.6012, *R* = 0.1470	*p* = 0.2696, *R* = 0.3047
Beta	*p* = 0.2274, *R* = 0.3315	*p* = 0.0985, *R* = −0.4427	*p* = 0.7076, *R* = 0.1057	*p* = 0.1944, *R* = −0.3548
Gamma	*p* = 0.3886, *R* = 0.2401	*p* = 0.1540, *R* = −0.3871	*p* = 0.1896, *R* = 0.3584	*p* = 0.5143, *R* = −0.1828
Cardiac sympathetic	*p* = 0.6147, *R* = −0.1416			
Cardiac vagal	*p* = 0.6981, *R* = 0.1093			

## DISCUSSION

The intricate interplay between the brain and heartbeat dynamics can dynamically change in response to specific cognitive and pathological conditions (Candia-Rivera, 2022; Samuels, 2007; Silvani et al., 2016). To comprehend these changes, methodological frameworks have been developed to study the role of brain–heart interactions. These frameworks rely on different signal processing techniques to quantify the presence of dynamic interactions between two physiological signals (Candia-Rivera, Catrambone, & Valenza, 2021). However, little is known about how brain network organization and heartbeats interact. Specifically, whether cardiac sympathetic–vagal dynamics may influence the large-scale neural organization in the brain remains to be tested. In this study, we aimed to provide a new framework to study brain–heart interactions in order to estimate brain network–cardiac couplings. For this analysis, we have considered network fluctuations, specifically on metrics quantifying clustering, efficiency, assortativity, and modularity, and their relationships with cardiac sympathetic–vagal activity.

We have analyzed brain network metrics from EEG data and cardiac sympathetic–vagal activity to compare healthy participants in resting state with high arousal emotion elicitation, and to compare the resting-state dynamics of patients with Parkinson’s disease on and off dopamine medication, with respect to the ones in healthy state. Our results on emotion elicitation showed a generalized increase in the network-cardiac couplings, with respect to resting state. These results indicate a high reactivity of the brain–heart interaction under the processing of affective states. The heartbeat dynamics reactivity for emotions has been shown in different studies (Pace-Schott et al., 2019), with a convergence to indicate emotion intensity and regulation (Candia-Rivera et al., 2022; Klein et al., 2021). It remains to be further explored whether the large-scale brain–heart relationships that can be identified with our framework are associated with any specific aspect of the emotional state.

Our results on the Parkinson’s disease dataset suggest that patients have distinct cortical network segregation that covaries with cardiac sympathetic–vagal activity. These covariations were confirmed by comparing Parkinson’s disease patients under their traditional treatments (on dopamine medication), which is mainly based on pharmacological dopamine replacement, against suppressed treatment for at least 12 hr (off dopamine medication). Parkinson’s disease patients off dopamine medication slowed their fluctuations in network segregation, and this slowing got in a certain degree of synchrony with cardiac sympathetic activity. We further showed that the increase in the network segregation–vagal coupling on dopamine medication related to improvements in motor symptoms. Our results indicate that dopaminergic therapy in Parkinson’s disease patients causes dynamic fluctuations in brain network organization, and these network dynamics are closely related to the autonomic nervous system. This new evidence suggests further links between known changes in brain networks (Conti et al., 2022; Hammond et al., 2007; Leviashvili et al., 2022) and autonomic dysfunction (Sharabi et al., 2021) that have been reported in Parkinson’s disease.

We confirmed that the changes observed in network-cardiac couplings were not exclusively triggered by changes in the EEG network. Instead, the changes appear as a result of synergistic phenomena that were quantified through the brain network–cardiac couplings. We controlled that when comparing the different conditions, the various network metrics exhibited lower statistical power in comparison to their respective brain–heart coupling counterparts. However, a previous study indicated that different network metrics could distinguish early-stage Parkinson’s disease patients from healthy controls (Conti et al., 2022), suggesting a potential relationship between dopaminergic levels and network organizations. These effects were likely induced by the variations in network densities (De Vico Fallani et al., 2017), which are known to occur in Parkinson’s disease patients owing to cortical hyper-synchronization (McCarthy et al., 2011). To address the bias caused by the differences in network densities, we utilized the efficiency–cost optimization algorithm (De Vico Fallani et al., 2017) to accurately analyze the redistribution of connections in the different conditions; we did not find significant differences between on- and off-dopamine medication conditions or with the changes in motor symptoms.

The interplay between the brain and peripheral bodily systems involves various neural pathways connected to the amygdala, thalamus, hypothalamus and prefrontal/frontal, insular, somatosensory, and cingulate cortices (Cameron, 2009). The existing methodological proposals to measure the brain–heart interaction include various signal processing methods, such as physiological modeling-based approaches, synchronization measurement, and transient neural responses to heartbeats (Candia-Rivera, Catrambone, & Valenza, 2021). Although the identification of individual brain regions in the control of peripheral organs has helped to define the components of the central autonomic network (Beissner, Meissner, Bär, & Napadow, 2013; Valenza, Passamonti, Duggento, Toschi, & Barbieri, 2020; Valenza et al., 2019), our framework can potentially reveal large-scale functional relationships linking brain network organization and autonomic dynamics. Furthermore, our results could provide new insights about the role of ascending signals in shaping integration and segregation in the brain (Shine, 2019). Through these efforts, we could draw relationships between specific brain oscillations and cardiac dynamics. Sympathetic and parasympathetic activities regulate heart rate dynamics for maintaining physiological balance, with the sympathetic system typically associated with preparing the body for action and the parasympathetic system with promoting relaxation and recovery (Porges, 2007). However, the intricacies between these systems and their connection to the brain under various conditions remain to be further described (Chen et al., 2021). On the brain side, for instance, a wide range of brain wave activities reflect different states of consciousness and cognitive engagement, with alpha typically associated with inhibition/activation mechanisms, beta with alertness and motor synergies, and gamma with active sensory processing (Cohen, 2017). By employing brain–heart interaction frameworks, we can provide a comprehensive description from a nervous-system-wise perspective. This approach may help us to better understand the specific connections and their functional roles.

Researching the brain–heart connection can yield valuable scientific discoveries and innovative clinical applications, particularly in regard to autonomic dysfunctions present after neurodegeneration (Iniguez et al., 2022; Sharabi et al., 2021). By these efforts, we may further uncover that heartbeat dynamics may contribute to conditions traditionally associated with the brain, and vice versa. For instance, recent findings have revealed the impact of cardiac factors on depression (Penninx, 2017) and motor function (Agrimi et al., 2023; Heimler et al., 2023). Numerous studies demonstrated the influence of heartbeat patterns on cognition (Azzalini et al., 2019; Candia-Rivera, 2022; Skora et al., 2022). It is important to investigate whether autonomic dysfunctions linked to certain diseases can affect neural dynamics at a larger scale. Specifically, Parkinson’s disease has shown large-scale, multisystem network disruptions (Gratton et al., 2019; Wang, Zhang, Lei, & Guo, 2021). The intricate communication between the brain and bodily systems highlights the significance of interoceptive mechanisms on neural homeostasis, and any dysfunctions that emerge may have neurological, psychiatric, or behavioral implications.

An important limitation of the study is the use of 32-channel EEG recordings. For this reason, we performed electrode-based connectivity and network analysis, instead of performing analyses on source-reconstructed signals. Nevertheless, our study may serve as a potential development of tools to be utilized in easily accessible clinical setups. The potential of EEG to comprehend the physiopathology of Parkinson’s disease is noteworthy, as it underscores the immense possibility of using EEG-based measurements to evaluate the disease and its comorbidities (George et al., 2013; Jackson, Cole, Voytek, & Swann, 2019; Swann et al., 2015). While changes in brain oscillations are potentially useful to evaluate dopamine medication effectiveness (Litvak et al., 2012), our results suggest that brain–heart interactions may better capture the improvement in motor symptoms triggered by dopaminergic replacement therapy. It is important to highlight that revising the upper limit of the gamma band could have a considerable impact on the calculation of metrics relating to the coupling between brain networks and cardiac activity (see the Supporting Information). As such, the choice of frequency band definitions should align with existing literature for each specific application.

Our framework unraveled large-scale dynamics into the connection between cortical connectivity and cardiac dynamics, shedding light on the network physiology in health and disease. These developments hold potential for the evaluation of cognitive and motor functions. By examining the relationship of cardiac dynamics with various properties of brain network organization, this framework may provide further insights into the role of the brain–heart network physiology.

## MATERIALS AND METHODS

### Emotion Elicitation Dataset

The dataset includes 32 healthy participants (median age 27 years, 16 males and 16 females). The data are part of the DEAP database for emotion analysis (Koelstra et al., 2012), available at https://www.eecs.qmul.ac.uk/mmv/datasets/deap/. Data were acquired using 32-channel EEG and three-lead ECG, sampled at 512 Hz. The dataset consisted of 40 video trials from music videos. Videos had a duration of 60 s and were presented after an initial resting period of 120 s.

The participants’ ratings of the emotional experience relied on the circumplex model of affect (valence related to pleasantness and arousal related to intensity). Prior research using this dataset has revealed that brain–heart interactions can function as biomarkers to distinguish between high and low arousal based on brain–heart interactions. (Candia-Rivera et al., 2022). Building upon those findings, we selected the trials based on group median arousal from the self-assessment scores. Group median arousal scores ranged between 3.2 and 7 among the 40 trials. We selected the high-arousal group with scores ranging 6.1–7 (11 trials).

### Parkinson’s Disease Dataset

The dataset includes 15 patients with Parkinson’s disease (7 males and 8 females, mean age = 63.2 ± 8.2 years) and 16 healthy participants (7 males and 9 females, median age = 60.5 ± 8 years). The data are part of a publicly available dataset, UC San Diego Resting State EEG Data from Patients with Parkinson’s Disease, gathered from OpenNeuro.org on November 21, 2022 (Appelhoff et al., 2019; Pernet et al., 2019). Participants provided written consent in accordance with the Declaration of Helsinki (Rockhill, Jackson, George, Aron, & Swann, 2021). Patients’ data were analyzed in the on-medication and off-medication conditions (discontinued medication use at least 12 hr before the session). Dopaminergic medication significantly improved motor symptoms, as measured by the motor section of the Unified Parkinson’s Disease Rating Scale Part III, UPDRS-III (Ramaker, Marinus, Stiggelbout, & van Hilten, 2002), as performed in a paired Wilcoxon test (*Z* = 2.9388, *p* = 0.0033).

EEG data were acquired using a 32-channel BioSemi ActiveTwo system, together with a one-lead ECG, sampled at 512 Hz at rest for approximately 3 min. During data collection, the participants were seated comfortably and told to fixate on a cross presented on a screen.

### EEG and ECG Data Processing

The EEG and ECG data were preprocessed using MATLAB R2022b and FieldTrip Toolbox (Oostenveld, Fries, Maris, & Schoffelen, 2011). The EEG and ECG data were bandpass filtered with a Butterworth filter of order 4 between 0.5 and 45 Hz. Large movement artifacts were removed from EEG using a wavelet-enhanced independent component analysis (ICA) (Gabard-Durnam, Mendez Leal, Wilkinson, & Levin, 2018). ICA was then rerun to detect and set to zero the components with eye movements and cardiac-field artifacts. One lead of ECG was included in the ICA computation to improve the process of identifying cardiac artifacts. EEG channels were re-referenced using a common average (Candia-Rivera, Catrambone, & Valenza, 2021).

The R-peaks from the ECG were identified using an automatized process, followed by an automated inspection of misdetections and manual correction if required. The procedure was based on a template-based method for detecting R-peaks (Candia-Rivera, Catrambone, & Valenza, 2021). For the correction of misdetection, all the detected peaks were visually inspected over the original ECG, along with the marks on potentially misdetected heartbeats and the interbeat interval (IBI) histogram.

### Computation of Cardiac Sympathetic and Vagal Indices

A method based on Poincaré plots was used for estimating cardiac sympathetic and vagal activities. The method uses the fluctuating geometry of the Poincaré plot constructed from IBI (Candia-Rivera, 2023). The Poincaré plot is a nonlinear approach used to analyze heart rate variability by illustrating fluctuations in the duration of consecutive IBIs (Brennan, Palaniswami, & Kamen, 2001; Woo, Stevenson, Moser, Trelease, & Harper, 1992). The SD1 and SD2 are calculated from the Poincaré plot and represent the short- and long-term fluctuations of heart rate variability, respectively. These values are obtained by determining the ratios of the ellipse formed by consecutive changes in IBIs (Sassi et al., 2015). The ellipse ratios for the whole experimental condition *SD*_01_ and *SD*_02_ are computed as follows:

$${SD}_{01}=\sqrt{\frac{1}{2}\;std{\left({IBI}^{\prime }\right)}^{2}},$$ (1)

$${SD}_{02}=\sqrt{2\;std{\left(IBI\right)}^{2}-\frac{1}{2}\;std{\left({IBI}^{\prime }\right)}^{2}},$$ (2)

where *IBI*′ is the derivative of IBI and *std*() refers to the standard deviation.

The time-varying fluctuations of the ellipse ratios are computed with a sliding time window, as shown in Equations 3 and 4:

$${SD}_{1}\left(t\right)=\sqrt{\frac{1}{2}\;std{\left({IBI}_{\Omega_{t}}^{\prime }\right)}^{2}},$$ (3)

$${SD}_{2}\left(t\right)=\sqrt{2\;std{\left({IBI}_{\Omega_{t}}\right)}^{2}-\frac{1}{2}\;std{\left({IBI}_{\Omega_{t}}^{\prime }\right)}^{2}},$$ (4)

where Ω_t_: *t* − *T* ≤ *t*_*i*_ ≤ *t*, and *T* is fixed in 15 s as proposed in previous simulation studies (Candia-Rivera, Catrambone, Barbieri, & Valenza, 2021).

The Cardiac Vagal Index (CVI) and Cardiac Sympathetic Index (CSI) are computed as follows:

$$CVI\left(t\right)={SD}_{01}+\bar{{SD}_{1}}\left(t\right),$$ (5)

$$CSI\left(t\right)={SD}_{02}+\bar{{SD}_{2}}\left(t\right),$$ (6)

where $\bar{{SD}_{x}}$ is the demeaned *SD*_*x*_.

Note that other measures of autonomic outflow can be used in our framework, such as oscillations gathered from standard low or high frequency (Orini, Bailón, Mainardi, Laguna, & Flandrin, 2012) or Laguerre expansions of heart rate variability series (Valenza, Citi, Saul, & Barbieri, 2018).

For a comprehensive description of the model, see Candia-Rivera (2023). The software can be gathered from https://github.com/diegocandiar/brain_heart_psv_sdg.

### Brain Network Construction and Metrics

EEG connectivity matrices were gathered from EEG spectral coherence (Carter, 1987). EEG power and cross-spectral densities were computed using the short-time Fourier transform with a Hanning taper. Calculations were performed through a sliding time window of 2 s with a 50% overlap. For each pair of EEG time series *x*_*i*_(*t*) and *x*_*j*_(*t*), and their respective complex Fourier transform *x*_*i*_[*f*] and *x*_*j*_[*f*], the coherence *COH*_*i*,*j*_[*f*] at the frequency *f* is defined as follows:

$${COH}_{i,j}\left[f\right]=\frac{\left|P_{i,j}\left[f\right]\right|}{{\left(P_{i}\left[f\right]\cdot P_{j}\left[f\right]\right)}^{0.5}},$$ (7)

where *P*_*i*,*j*_[*f*] is the cross-spectrum of *x*_*i*_[*f*] and *x*_*j*_[*f*] and *P*_*n*={*i*,*j*}_[*f*] is the power spectral density of *x*_*n*={*i*,*j*}_(*t*). The output’s absolute value was considered for the network analyses. Coherence matrices were integrated within three frequency bands (alpha: 8–12 Hz, beta: 12–30 Hz, gamma: 30–45 Hz), based on previous findings on cortical connectivity in Parkinson’s disease (Conti et al., 2022). The connectivity matrices were filtered using the efficiency–cost optimization algorithm to obtain binary and undirected connectivity graphs (De Vico Fallani et al., 2017).

Different network metrics were computed per each connectivity graph. Network metric calculations were performed in each connectivity sample, resulting in fluctuating network characterizations with 1-s resolution. In this study we have focused on global network metrics: clustering, efficiency, assortativity, and modularity. Efficiency is a measure that indicates the amount of information by assuming that the less connected the nodes are in the network, the less efficient their communication is (Latora & Marchiori, 2001). Therefore, efficiency can be considered a measure of network integration. Efficiency was computed as shown in Equation 8:

$$E=\frac{1}{n}\;\sum_{i\in N}E_{i}=\frac{1}{n}\;\sum_{i\in N}\frac{\sum_{j\in N,j\neq i}{d_{ij}}^{-1}}{n-1},$$ (8)

where *n* is the number of nodes in the network and *d*_*ij*_ is the distance between the nodes *i* and *j*. In this study, the connectivity matrices are binary and the distance between the nodes is quantified by the shortest path.

Clustering was measured using network transitivity, a measure that indicates the tendency of the nodes of the network to be grouped, meaning that a high transitivity indicates that the network contains a high amount of groups of nodes, based in their connectivity (Humphries & Gurney, 2008; Newman, 2003). Transitivity was computed as shown in Equation 9:

$$T=\frac{Tr\left(a^{3}\right)}{\sum_{i,j,k\in N}a_{ij}a_{jk}a_{ki}},$$ (9)

where *Tr* is the trace function and *a*_*ij*_ is a connectivity value between the nodes *i* and *j*.

Assortativity is a measure that indicates the preference of the nodes of the network to be connected with other nodes with similar connectivity (Newman, 2002). Assortativity was computed as shown in Equation 10:

$$A=\frac{M^{-1}\;\sum_{i}j_{i}\;k_{i}-{\left[M^{-1}\;\sum_{i}0.5\;\left(j_{i}+k_{i}\right)\right]}^{2}}{M^{-1}\;\sum_{i}0.5\;\left({j_{i}}^{2}+{k_{i}}^{2}\right)-{\left[M^{-1}\;\sum_{i}0.5\;\left(j_{i}+k_{i}\right)\right]}^{2}},$$ (10)

where *j*_*i*_, *k*_*i*_ are the degrees of the vertices at the ends of the *i*th edge, with *i* = 1, …, *M*.

Modularity is a measure that indicates the network’s amount of division into groups (modules). High modularity indicates a dense connection between the nodes within groups and sparse connections between nodes in different groups (Newman, 2006). Modularity was computed as shown in Equation 11:

$$Q=\frac{1}{2L}\sum_{i,j\in N}\left[a_{ij}-\frac{k_{i}k_{j}}{2L}\right]\delta \left(s_{i},s_{j}\right),$$ (11)

where *L* is the sum of all connectivity values, *a*_*ij*_ is a connectivity value between the nodes *i* and *j*, *k*_*i*_ is the degree of node *i*, and *s*_*i*_ indicates the group to which node *i* belongs. The term *δ*(*s*_*i*_, *s*_*j*_) is the Kronecker delta function, which is equal to 1 if *s*_*i*_ = *s*_*j*_, and 0 otherwise. The optimal definition of groups or community structures were defined as network subdivisions with nonoverlapping groups of nodes, in which the number of within-group edges is maximized, and the number of between-group edges is minimized.

The computation of the network metrics was performed per each time stamp, to gather a time-varying estimation of the global network organization. The network metrics were computed using the Brain Connectivity Toolbox (Rubinov & Sporns, 2010) and followed the default procedures defined in the toolbox. Note that further network metrics can be analyzed under this framework, such as clustering coefficient (Watts & Strogatz, 1998), closeness centrality (Freeman, 1978), or betweenness centrality (Freeman, 1978).

### Brain Network–Cardiac Coupling Estimation

To quantify the coupling between the fluctuations of brain network metrics and cardiac dynamics, we used the maximal information coefficient (MIC). MIC is a method that quantifies the coupling between two time series (Reshef et al., 2011). MIC may capture nonlinear correlations, as it considers the similarities between two time series irrespective of signal curvatures. MIC evaluates similarities between different segments separately at an adapted time scale that maximizes the mutual information, with a final measure that wraps the similarities across the whole time course. Equations 12 and 13 show the MIC computation between two time series X and Y. The mutual information *I*_*g*_ is computed for different grid combinations *g* ∈ *G*_*xy*_. The mutual information values are normalized by the minimum joint entropy log_2_ min {*n*_*x*_, *n*_*y*_}, resulting in an index in the range 0–1. Finally, the quantified coupling between X and Y corresponds to the normalized mutual information resulting from the grid that maximizes the MIC value.

$$\text{m}\left(\text{X},\text{Y}\right)=\frac{\max_{g\in G_{xy}}\;I_{g}}{\log_{2}\min\left\{n_{x},n_{y}\right\}},$$ (12)

$$\text{MIC}\left(\text{X},\text{Y}\right)=\max_{n_{x}\times n_{y}<B}m\left(X,Y\right),$$ (13)

where *B* = *N*^0.6^, and *N* is the dimension of the signals (Reshef et al., 2011). The source code implementing MIC is available online at https://github.com/minepy.

### Statistical Analysis

Statistical comparisons were based on Wilcoxon tests and Spearman correlations. Wilcoxon signed rank tests for paired comparisons were performed between the biomarkers comparing resting state and emotion elicitation in healthy participants, and Parkinson’s disease in the on- and off-dopamine medication conditions. Wilcoxon rank sum tests for unpaired comparisons were performed between the biomarkers comparing healthy participants and Parkinson’s disease patients. Spearman correlation coefficients were calculated for the changes in motor symptoms (quantified with the UPDRS-III scale) and the changes in network-cardiac coupling. *P* values associated with the Spearman correlation coefficients were derived by a *t* Student distribution approximation. Significance of the statistical tests was considered at α = 0.05. To assess the significance of the comparisons that resulted in *p* < 0.05, we performed Monte Carlo permutations to reject the hypothesis of a random effect. Monte Carlo *p* values (*p*_mc_) were calculated by determining the proportion of permutations that exceeded the original statistical significance, out of 1,000 random permutations (Maris & Oostenveld, 2007). Significance was then confirmed if *p*_mc_ < 0.05.

## SUPPORTING INFORMATION

Supporting information for this article is available at https://doi.org/10.1162/netn_a_00369.

## AUTHOR CONTRIBUTIONS

Diego Candia-Rivera: Conceptualization; Formal analysis; Investigation; Methodology; Software; Visualization; Writing – original draft; Writing – review & editing. Mario Chavez: Formal analysis; Funding acquisition; Supervision; Writing – review & editing. Fabrizio De Vico Fallani: Formal analysis; Funding acquisition; Supervision; Writing – review & editing.

## FUNDING INFORMATION

Fabrizio De Vico Fallani, Agence Nationale de la Recherche (https://dx.doi.org/10.13039/501100001665), Award ID: ANR-20-CE37-0012-03.

## Supplementary Material



## TECHNICAL TERMS

Network efficiency: A measure of how much a network shares information within all its nodes.
Network clustering (or transitivity): A measure of how much the connections of a network tend to form clusters.
Network modularity: A measure of how much a network is divided into densely connected modules.
Network assortativity: A measure of how much a network tends to preferentially connect its nodes to others with similar degrees.
Coherence: A measure of linear correlation between two signals, revealing their synchronization in their cross-spectral densities, accounting for phase and amplitude matches.
Poincaré plot: A method for visualizing and analyzing the variability of a signal by contrasting its consecutive changes.
Maximal information coefficient: A measure of linear and nonlinear associations between two signals, based on their mutual information and joint entropy.
Mutual information: A measure of the mutual dependence between two signals, capturing shared information and associations.
Joint entropy: A measure of uncertainty in a system by accounting for the total information content.
