# Supplementary material for: Measures of the coupling between fluctuating brain network organization and heartbeat dynamics
Source: Netw Neurosci. 2024 Jul 1;8(2):557–75. doi: 10.1162/netn_a_00369 (PMC11168717; doi:10.1162/netn_a_00369)
Supplement: Supplementary file 1 [file netn-8-2-557-s001.docx]

Measures of the coupling between fluctuating brain network organization and heartbeat dynamics

Diego Candia-Rivera*, Mario Chavez, and Fabrizio de Vico Fallani

*Sorbonne Université, Paris Brain Institute (ICM), CNRS UMR7225, INRIA Paris (Nerv Team), INSERM U1127, AP-HP Hôpital Pitié-Salpêtrière, F75013, Paris, France*

** Correspondence: diego.candia.r@ug.uchile.cl*

*
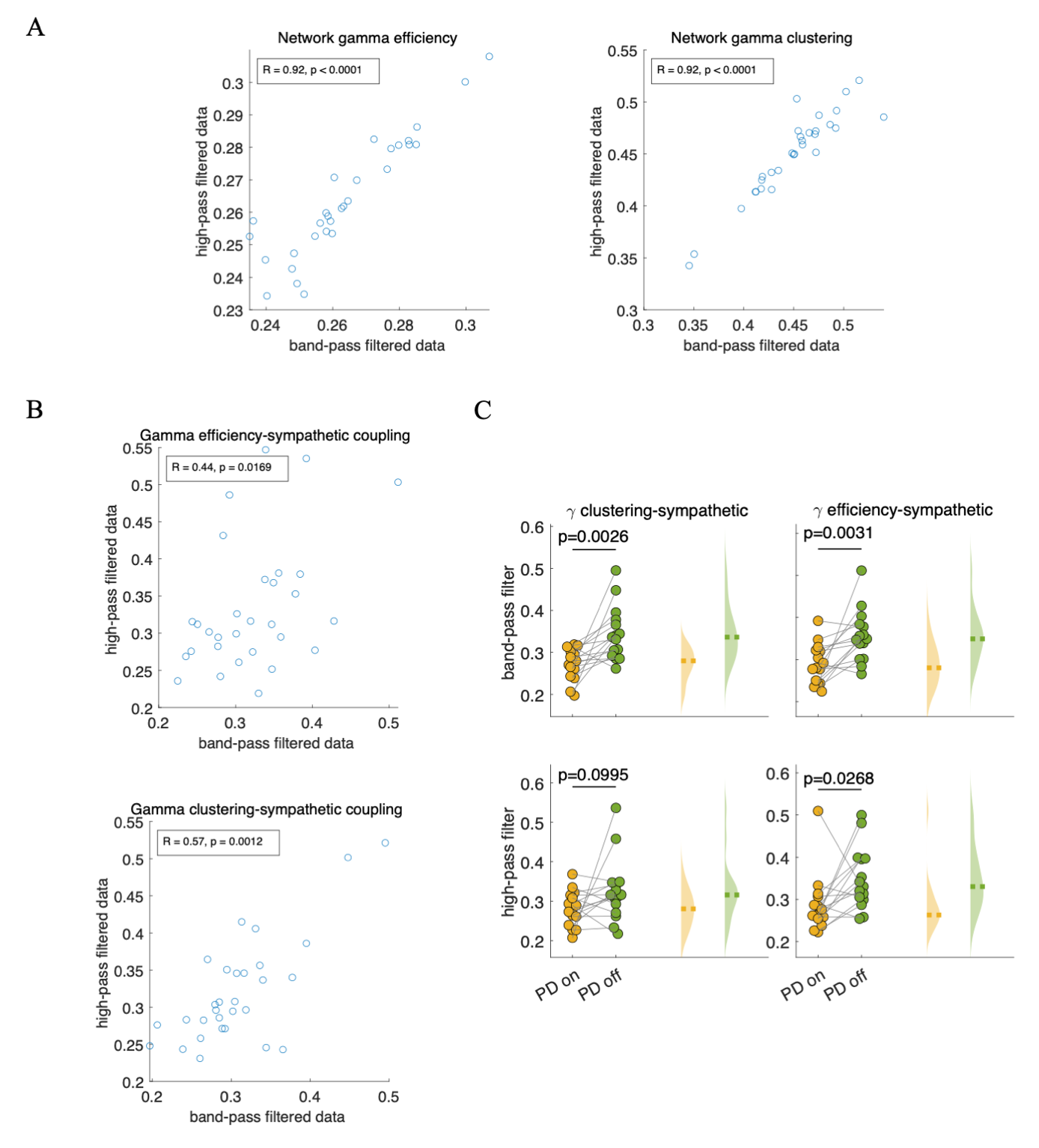
*

*Figure S1. Effects of the preprocessing filter on the brain network-cardiac coupling in the gamma band (30-45 Hz). The filters compared are a bandpass filter between 1-45 Hz and a high pass filter at 1 Hz. (A) Correlation analysis on gamma clustering and gamma efficiency. Each data point corresponds to one Parkinson’s disease patient, x-axis to the preprocessing including a band-pass filter and the y-axis the high-pass filter. The conditions on and off dopamine medication were pooled together. Both network measures resulted correlated with a correlation coefficient R = 0.92 (p < 0.0001). (B) Correlation analysis on gamma clustering-sympathetic and gamma efficiency-sympathetic coupling. Each data point corresponds to one Parkinson’s disease patient, x-axis to the preprocessing including a band-pass filter and the y-axis the high-pass filter. The conditions on and off dopamine medication were pooled together. Both network measures resulted correlated with a correlation coefficient R = 0.44 (p = 0.0169) and R = 0.57 (p = 0.0012) for efficiency and clustering, respectively. (C) Paired Wilcoxon tests comparing the changes in gamma clustering-sympathetic and gamma efficiency-sympathetic couplings in Parkinson’s disease patients, on and off dopamine medication. The test were performed for the datasets that were band-pass and high-pass filtered, showing both the same trend with a higher statistical separability for the case of the band-pass filtering.*

**Table S1. Effect magnitudes (Z-values) from the brain-heart coupling measures that resulted significant when comparing the different conditions studied. The displayed effect magnitudes are accompanied to their respective brain and heart-only estimation.**

| **Conditions compared** | **Brain-heart component** | | | **Effect magnitude (Z-value)** | | |
| --- | --- | --- | --- | --- | --- | --- |
|  |  |  |  | **Brain-heart coupling** | **Brain only** | **Heart only** |
| **Rest vs Emotion elicitation** | **Cardiac part** | **Brain part** | |  |  |  |
|  | **Sympathetic** | **Alpha** | **Clustering** | **3.7211** | 1.0471 | **3.5341** |
|  |  |  | **Efficiency** | **3.5715** | -0.6171 | **3.5341** |
|  |  |  | **Assortativity** | **3.7211** | 0.6171 | **3.5341** |
|  |  |  | **Modularity** | 3.4220 | -0.2618 | **3.5341** |
|  |  | **Beta** | **Clustering** | 3.4406 | 1.4024 | **3.5341** |
|  |  |  | **Efficiency** | 3.0853 | -1.3463 | **3.5341** |
|  |  |  | **Assortativity** | **3.9642** | -0.3740 | **3.5341** |
|  |  |  | **Modularity** | **3.7772** | 0.1870 | **3.5341** |
|  |  | **Gamma** | **Clustering** | **3.7772** | 0.1122 | **3.5341** |
|  |  |  | **Efficiency** | 2.9170 | -0.3366 | **3.5341** |
|  |  |  | **Assortativity** | 3.2536 | -0.3366 | **3.5341** |
|  |  |  | **Modularity** | 3.4032 | -0.4862 | **3.5341** |
|  | **Parasympathetic** | **Alpha** | **Clustering** | **3.3284** | 1.0471 | **3.1601** |
|  |  |  | **Efficiency** | **4.4129** | -0.6171 | **3.1601** |
|  |  |  | **Assortativity** | **4.0764** | 0.6171 | **3.1601** |
|  |  |  | **Modularity** | **4.5251** | -0.2618 | **3.1601** |
|  |  | **Beta** | **Clustering** | **4.1699** | 1.4024 | **3.1601** |
|  |  |  | **Efficiency** | 2.7861 | -1.3463 | **3.1601** |
|  |  |  | **Assortativity** | **3.7959** | -0.3740 | **3.1601** |
|  |  |  | **Modularity** | **3.3471** | 0.1870 | **3.1601** |
|  |  | **Gamma** | **Clustering** | **4.3382** | 0.1122 | **3.1601** |
|  |  |  | **Efficiency** | **3.9648** | -0.3366 | **3.1601** |
|  |  |  | **Assortativity** | **3.7211** | -0.3366 | **3.1601** |
|  |  |  | **Modularity** | 2.6552 | -0.4862 | **3.1601** |
| **PD on vs PD off** | **Sympathetic-alpha clustering** | | | **2.2151** | -0.6248 | 0.3976 |
|  | **Sympathetic-gamma clustering** | | | **3.0102** | -1.3631 | 0.3976 |
|  | **Sympathetic-gamma efficiency** | | | **2.9534** | 1.3063 | 0.3976 |
| **Healthy state vs PD off** | **Sympathetic-alpha modularity** | | | **2.1148** | 1.2056 | -0.6522 |
|  | **Sympathetic-gamma efficiency** | | | **2.1543** | -1.3637 | -0.6522 |

**Bold indicates that brain-heart coupling effect magnitude is greater than the effect magnitudes observed in both, brain-only and heart-only comparisons.**

**Table S2. Median ± Median absolute deviation (MAD) of the difference between “on” and “off” dopamine in PD patients, for the markers of brain-heart coupling that resulted significant in the main results, and their respective brain network and heart counterparts. P-values of the Wilcoxon tests are reported for to test whether the effect measured in the Δ Brain-heart coupling is larger than the ones measured in the respective Δ Brain network and Δ Heart.**

|  |  | **Δ Brain-heart coupling (median ± MAD)** | **Δ Brain network (median ± MAD)** | p-value | **Δ Heart (median ± MAD)** | p-value |
| --- | --- | --- | --- | --- | --- | --- |
| **PD on vs PD off** | **Sympathetic-alpha clustering** | 0.0321 **± 0.0242** | 0.0029 **± 0.0182** | **0.5614** | **0.0336 ± 0.1267** | 0.7197 |
|  | **Sympathetic-gamma clustering** | 0.0589 **± 0.0431** | 0.0104 **± 0.0276** | ****0.0125**** | **0.0336 ± 0.1267** | 0.3028 |
|  | **Sympathetic-gamma efficiency** | 0.0367 **± 0.0508** | 0.0013 **± 0.0099** | ****0.0020**** | **0.0336 ± 0.1267** | 0.3894 |

**Bold indicates p < 0.05**
